# New rapid one-step PCR diagnostic assay for *Plasmodium falciparum* infective mosquitoes

**DOI:** 10.1038/s41598-018-19780-6

**Published:** 2018-01-23

**Authors:** Mary Kefi, Konstantinos Mavridis, Maria L. Simões, George Dimopoulos, Inga Siden-Kiamos, John Vontas

**Affiliations:** 1 Institute of Molecular Biology and Biotechnology, Foundation for Research and Technology-Hellas, Heraklion, 70013 Greece; 2 Department of Biology, University of Crete, VassilikaVouton, Heraklion, 70013 Greece; 3 W. Harry Feinstone Department of Molecular Microbiology and Immunology, Johns Hopkins Bloomberg School of Public Health, 615N Wolfe St, Baltimore, MD 21205 USA; 4 Pesticide Science Laboratory, Department of Crop Science, Agricultural University of Athens, 11855 Athens, Greece

## Abstract

An essential component of malaria vector control programmes is the detection of *Plasmodium falciparum* within its mosquito vectors, particularly in the salivary glands where the infective sporozoites reside. Several protocols have been developed for this purpose; however they require dissection of mosquito specimens prior to analysis. Here, a novel one-step RT-qPCR TaqMan diagnostic assay was developed for mosquitoes with infective *Plasmodium falciparum* sporozoites in the salivary glands. It is based on detection of the sporozoite-specific Pf*slarp* and Pf*plp1* gene transcripts. These transcripts were chosen based on bioinformatics analysis, and experimentally verified to be overexpressed in the salivary gland sporozoite stage of the parasite compared to other mosquito parasite stages. The proof of principle and the performance of the assay were demonstrated using RNAlater preserved mosquito samples. Tests of analytical sensitivity showed the novel TaqMan assay to be 100% accurate, although its performance in the field needs to be further demonstrated. This method has no requirement for dissection and post-PCR processing and thus is simple and rapid to perform in individual mosquitoes or mosquito pools. It can be used in single or multiplex formats also targeting additional markers expressed in different tissues, such as detoxification enzymes associated with insecticide resistance.

## Introduction

Malaria is considered to be one of the most severe infectious diseases worldwide, causing about half a million deaths every year, primarily in the developing world^1^. It is transmitted by *Plasmodium*-infected female Anophelinae mosquitoes. Of the five *Plasmodium* species that infect humans, *Plasmodium falciparum* is the most important^2,3^. When a female *Plasmodium*-infected mosquito takes a blood meal, parasites (sporozoites) are transmitted to the vertebrate host through the injected saliva. After a brief developmental stage in the liver, the parasites are released into the blood stream where the asexual replicative cycles in the erythrocyte cause the pathology of the disease. Male and female gametocytes are formed in the erythrocytes, but the sexual stage is completed only after ingestion of a blood meal by the mosquito. This results in the formation of motile zygotes, which develop into ookinetes, which traverse the midgut epithelium and then transform into oocysts. In the oocyst, during approximately the next 10 days, sporozoites are formed, and when mature they are released into the hemolymph. The sporozoites will then translocate to the salivary glands where they are stored until an infective bite^4–6^. During their development in the mosquito the parasites suffer severe losses at different stages that are considered bottlenecks of the *Plasmodium* life cycle^5,7^. Hence not all gametocytes ingested by the mosquito result in sporozoite-infected salivary glands, and a mosquito is only considered infective when the sporozoites have invaded the salivary gland^8^.

Insecticide-treated nets (ITNs), indoor residual spraying (IRS), chemoprevention in pregnant women and children, artemisinin-based combination treatment (ACT), as well as human and vector diagnostic tests contributed to a great extent to the reduction of malaria in the last two decades^9^. However, the elimination of the threat from the countries where malaria is transmitted remains a challenge.

Prevention of malaria is best achieved by vector control, which today in Africa relies on the use of insecticides. Monitoring mosquito vector populations is an integral and essential component of most vector control programmes. Contemporary data on mosquito species composition, resistance to insecticides, and actual infection status of mosquito vectors, are a prerequisite for effective interventions^10^. The infection status probably represents the most important information, given the number of tests performed in the frame of country level monitoring activities. To date, the methods used for detecting infective vectors are labour intensive and/or have sensitivity and specificity issues. Dissection of salivary glands followed by microscopic examinations, or ELISA using an antibody against the Circumsporozoite Protein (CSP) present on sporozoites, has been used extensively for the identification of the epidemiologically relevant sporozoite-infected mosquitoes^11–13^. However, these assays require experienced personnel, particularly for mosquito dissections, while they also have sensitivity and specificity issues, due to hemolymph stage parasite contamination in the specimens that are examined, and often result in overestimation of infective mosquito rates. PCR-based molecular assays for the detection of *Plasmodium* in mosquitoes, such as nested PCR approaches^14^, are also used, as well as TaqMan diagnostic DNA-based assays, which require no post-PCR processing and can discriminate *P. falciparum* from *P. vivax*, *P. ovale* and *P. malariae*^15^. However, the latter method is based on DNA detection that also requires the dissection of the mosquito head and thorax and the removal of the abdomen prior to DNA extraction, in order to detect infective mosquitoes^15^. This limits its practicality, particularly when large pools of mosquito samples have to be tested, which is often necessary in low transmission settings.

In this study we identified salivary gland sporozoite-specific *Plasmodium* transcripts, which were subsequently used for the development of a specific diagnostic test for detecting mosquitoes carrying infective *P. falciparum* in their salivary glands.

## Materials and Methods

### Ethics Statement

All work was carried out in strict accordance with Greek regulations consisting of the Presidential Decree (160/91) and law (2015/92) which implement the directive 86/609/EEC from the European Union and the European Convention for the protection of vertebrate animals used for experimental and other scientific purposes and the new legislation Presidential Decree 56/2013. The experiments were carried out in a certified animal facility with the license (EL91-BIOexp-02) and the protocol has been approved by the FORTH Committee for Evaluation of Animal Procedures (6740/8/10/2014) and by the Prefecture of Crete (license number # 27290, 15/12/2014). This study was carried out in strict accordance with the recommendations in the Guide for the Care and Use of Laboratory Animals of the National Institutes of Health. The protocol was approved by the Animal Care and Use Committee of the Johns Hopkins University (permit number MO15H144). Commercial anonymous human blood was used for parasite cultures and mosquito feeding, and informed consent was therefore not applicable. The Johns Hopkins School of Public Health Ethics Committee has approved this protocol.

#### *Plasmodium berghei* life cycle

*Anopheles gambiae* G3 strain mosquitoes were raised at 28 °C and 80% humidity with a 12 h light/12 h dark cycle and maintained on a 10% sucrose solution during adult stages. 5-6-day old female mosquitoes were blood-fed on anesthetized adult *Mus musculus* mice that had been infected with *Plasmodium berghei*, strain ANKA 2.34. Rodents were assayed for high levels of parasitemia and for the abundance of gametocyte-stage parasites capable of exflagellation. After the infective blood meal, mosquitoes were maintained at 19 °C. On day 11 post-feeding, mosquitoes which had well developed ovaries were dissected in PBS and midguts were isolated and stored at −80 °C. Salivary glands were isolated on day 21 post-bloodmeal (pbm).

### RNA isolation from different stages of *Plasmodium berghei* and cDNA synthesis

RNA from the dissected midguts and salivary glands was isolated using Trizol reagent protocol (Ambion) following manufacturer’s instructions. DNase treatment followed by purification using Qiagen RNeasy MiniElute Cleanup Kit was done. For cDNA synthesis, 1 μg οf RNA was reverse transcribed using Thermoscript RT-PCR system kit (Invitrogen), with oligonucleotide primers and following manufacturer’s instructions. To confirm that cDNA was successfully synthesized, PCR was performed using primer pairs specific for RPS7 (*A. gambiae* Ribosomal Protein 7). Amplified DNA products were run on 1% agarose gels which were then stained with ethidium bromide. DNA size markers were either phage lambda DNA digested with StyI or 100 bp ladder from Solis BioDyne. Gel images were captured on the Gel Doc XR+imaging system (BioRad).

### *Plasmodium falciparum*-infected mosquitoes

*Anopheles gambiae* Keele strain mosquitoes were fed on a NF54 (MR4) *P. falciparum* gametocyte culture through artificial membranes at 37 °C and kept at 27 °C after the blood meal. The median number of oocysts/midgut was 2 (range of 0-42 oocysts/midgut) for a subset of mosquitoes (N = 22) on day 7 post-gametocyte ingestion. Mosquitoes were collected at 12 and 21 days pbm in RNAlater (Invitrogen), a reagent that stabilizes RNA in tissues. Samples were shipped at ambient temperature.

### RNA isolation from different stages of *Plasmodium falciparum* and cDNA synthesis

RNA from whole mosquitoes was isolated using PicoPure kit (Arcturus) according to the manufacturer’s instructions. After the extraction, DNase treatment and cDNA synthesis were carried out as described above.

### Comparison of expression levels between midgut and salivary gland sprorozoites in *Plasmodium berghei* and *Plasmodium falciparum*

Initially, primer pairs for every gene were designed and PCR conditions were optimized using gradient PCR (Supplementary Tables S1 and S2). Polymerase chain reaction (PCR) using GoTaq Pro polymerase (Promega) was carried out as described by the manufacturers in a BioRad thermal cycler. For testing each primer pair, a positive control containing genomic parasite DNA as template and a negative control without template were included in each experiment. For the five first *P. berghei* genes tested (Pb*slarp*, Pb*gest*, Pb*plp1*,Pb*spatr*, Pb*spect1*), 35 PCR cycles were used. For the remaining *P. berghei* genes and for all *P. falciparum* genes tested, samples were removed after 25, 30 and 35 PCR cycles. Amplified DNA products were detected as described above.

### Real-time RT-qPCR of *P. falciparum-*infected mosquitoes

Total RNA was extracted from mosquito pools 12- and 21- days post-infection (N = 5 pools of 10 individuals, from each stage) using TRI Reagent™ Solution (Invitrogen, Carlsbad, CA) according to the manufacturer’s instructions. Total RNA concentration was determined by spectrophotometry using a NanoDrop 2000c spectrophotometer (Thermo Scientific) and its integrity was assessed via agarose gel electrophoresis (1.0% w/v). A quantitative Reverse Transcription-real-time PCR (RT-qPCR) assay, based on TaqMan® chemistry, was designed and developed for the quantification of Pf*slarp* (accession no. XM_001348111.1) and Pf*plp1* (accession no. XM_001349297.1) target genes, normalized to Pf*csp* reference gene expression (accession no. XM_001351086.1). Gene-specific primers and probes were designed using the Primer Express software v 3.01 (Applied Biosystems, Foster City, CA). For the target genes, one primer spanned two exons in order to avoid DNA amplification. Each probe was labelled with a different fluorescent dye in order to evaluate the possibility of multiplexing. The analytical parameters of the RT-qPCR reactions are presented in Table 1. Reactions were performed in the ViiA 7 Real-Time PCR System (Applied Biosystems, Waltham, MA USA) using a one-step RT-PCR mastermix supplied by FTD (Fast Track Diagnostics, Luxembourg) and total RNA of at least 500 ng per sample in a total reaction volume of 10 μL. The thermal cycle parameters were: 50 °C for 15 min, 95 °C for 3 min, and 40 cycles of 95 °C for 3 sec and 60 °C for 30 sec, allowing a sample to result time of ~75 min. Samples were amplified in triplicates and each run always included a non-template control. *P. falciparum* DNA was also tested and no detectable signal was observed for the target genes. Additionally, both DNase and non-DNase treated aliquots of the same samples were assessed and no difference was observed in normalized expression levels (data not shown). The comparative Ct method was used for the calculation of Relative Quantification (RQ) units for each sample and each target gene (RQ = 2^−dCt^, where dCt = (Ct target - Ct reference)). The software package for calculating sensitivity and specificity was MedCalc the comparison of expression levels between the two groups was performed with the independent samples t-test using SPSS v17.0. In order to test the sensitivity of Pf*plp1* and Pf*slarp* detection in mosquito pools we mixed infected with non-infected *A. gambiae* mosquitoes in ratios of 1:10, 1:50 and 1:100 using two biological replicates per ratio. The previously described protocol for RT-qPCR was followed with the only modification being the use of 5 μg total RNA template for each sample. This study was performed according to the MIQE guidelines (Supplementary Table S3)^16^.

**Table 1:** Analytical parameters of the RT-qPCR reactions.

Reaction parameters				
Gene	Forward primer (5′-3′) (concentration in nM)	Reverse primer (5′-3′) (concentration in nM)	Probe (concentration in nM)	Amplicon size(bp)
Pf*slarp*	CCAAACACTCAGCACAGGAACA (500)	CCATACAGCCCTGGTATATAAATTA CTG (500)	FAM-ATGTCTATTGGCACTTACT- MGB (300)	131
Pf*plp1*	CCTTTTAGGGTTTGGTATATCCTC TTC (200)	GAGCAGCTTTTCATTCCTGGT (200)	HEX-TCAGGGAGAATCAATTC- MGB (250)	96
Pf*csp*	TCAACTGAATGGTCCCCATGT (100)	GAGCCAGGCTTTATTCTAACTTGAA T (200)	Cy5-TGTAACTTGTGGAAATGG- MGB-(250)	68
**Quality control**				
**Gene**	**Reaction Efficiency (%)**	**Linearity (R** ^**2**^ **)**	**Dynamic Range (Ct)**	
Pf*slarp*	108.5	0.9992	24.0–34.0	
Pf*plp1*	99.70	0.9969	26.0–36.0	
Pf*csp*	95.5	0.9996	16.0–30.0	
**% CV of RQ units**		**Multiplexing**		
Pf*slarp*	19.9	2-plex (A): Pf*plp1* + Pf*csp*	2-plex (B): Pf*slarp* + Pf*csp*	
Pf*plp1*	9.8	No change in sensitivity	Loss of sensitivity for Pf*slarp* (+1.5 Ct shift)	

### Data availability statement

Materials, data and associated protocols are available upon request.

## Results

### Identification of salivary gland-specific *Plasmodium berghei* sporozoite transcripts

**Figure 1:**
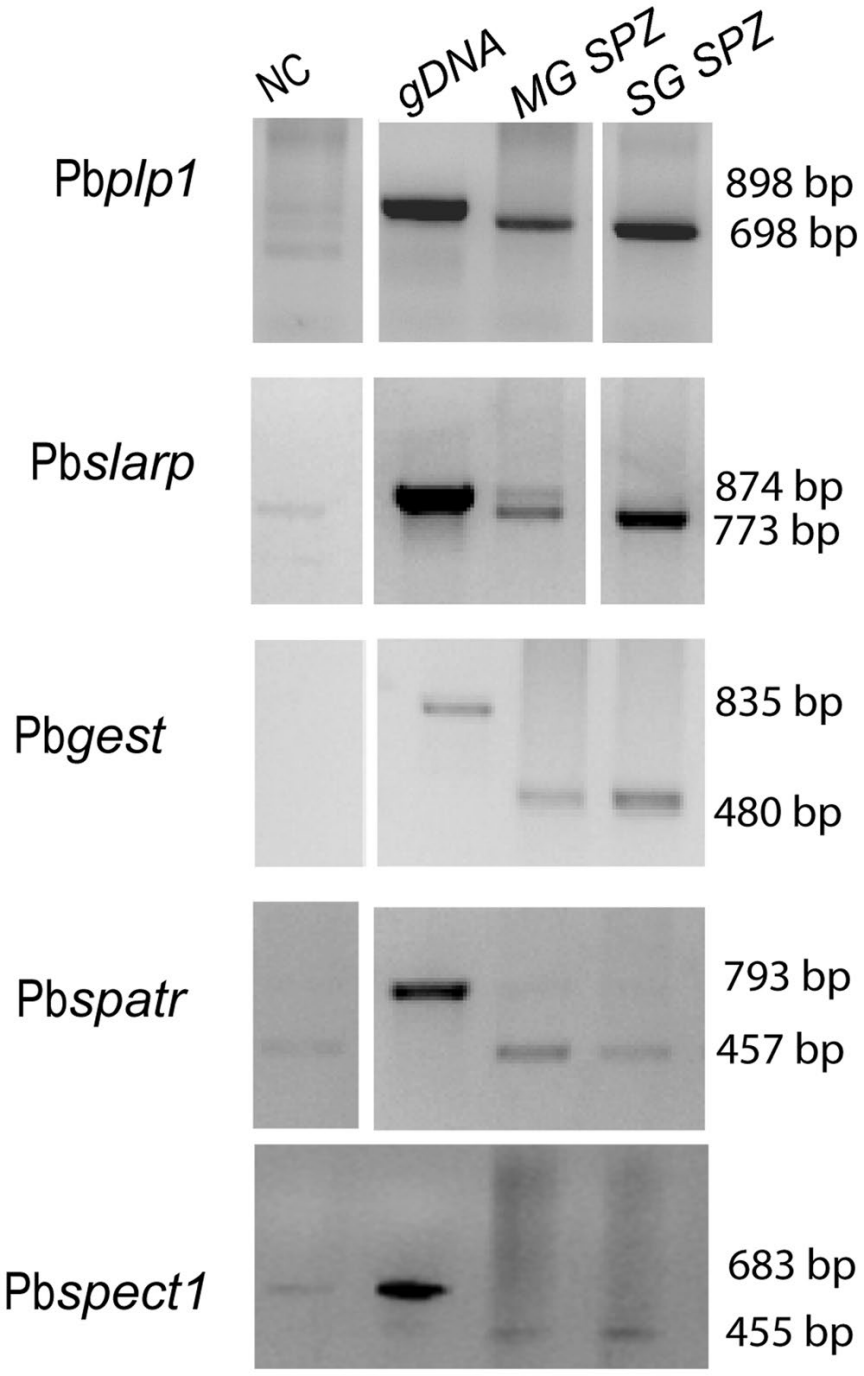
RT-PCR analysis of *P.berghei* Pb*plp1*, Pb*gest*, Pb*spectI*, Pb*spatr* and Pb*slarp* transcripts. Templates were derived from gDNA (lane 2), dissected midguts at 11 d pbm (post blood meal) midgut sporozoites (lane 3, MG SPZ) and dissected salivary gland sporozoites at 21 d pbm (lane 4 SG SPZ). Lane 1 is the negative control without template (NC). The samples were amplified for 35 cycles. Molecular weight of the amplified fragments is indicated to the right. Unprocessed images of the agarose gels are shown in Supplementary Fig. S4a.

As an initial test for the identification of a transcriptional marker for salivary gland-stage sporozoites, we used the rodent model parasite *P. berghei*. Due to the absence of transcriptomic data for the stages of interest we used Plasmodb proteomic data (www.Plasmodb.org) to identify candidate genes. Five genes were selected: Pb*plp1* (perforin-like protein 1, also called Pb*spect2*, PBANKA_1006300), Pb*gest* (gamete egress and sporozoite traversal protein, PBANKA_1312700), Pb*spatr* (secreted protein with altered thrombospondin repeat domain, PBANKA_0309500), Pb*slarp* (sporozoite and liver-stage asparagin-rich protein, PBANKA_0902100) and Pb*spect1* (sporozoite micronemal protein essential for cell traversal, PBANKA_1355600). For the preparation of the tissue-specific templates, midguts and salivary glands were isolated from *A. gambiae* mosquitoes, previously fed on *P. berghe*i-infected mice. Midguts were dissected on day 11and salivary glands on day 21 post-infectious blood meal. The presence of oocysts (2 to 10 per infected mosquito) was confirmed in mosquito guts in parallel infections. Semi-quantitative reverse transcribed PCR, using specific gene primers, was then carried out. These experiments revealed that the transcript levels of Pb*gest*, Pb*plp1* and Pb*slarp* were higher in salivary gland sporozoites, but they were also detected in the midgut samples (Fig. 1). The discrepancy with the proteomic results suggests that the expression might be regulated post-transcriptionally, although technical/sensitivity issues that may have restricted the proteomic analysis cannot be excluded.

**Figure 2:**
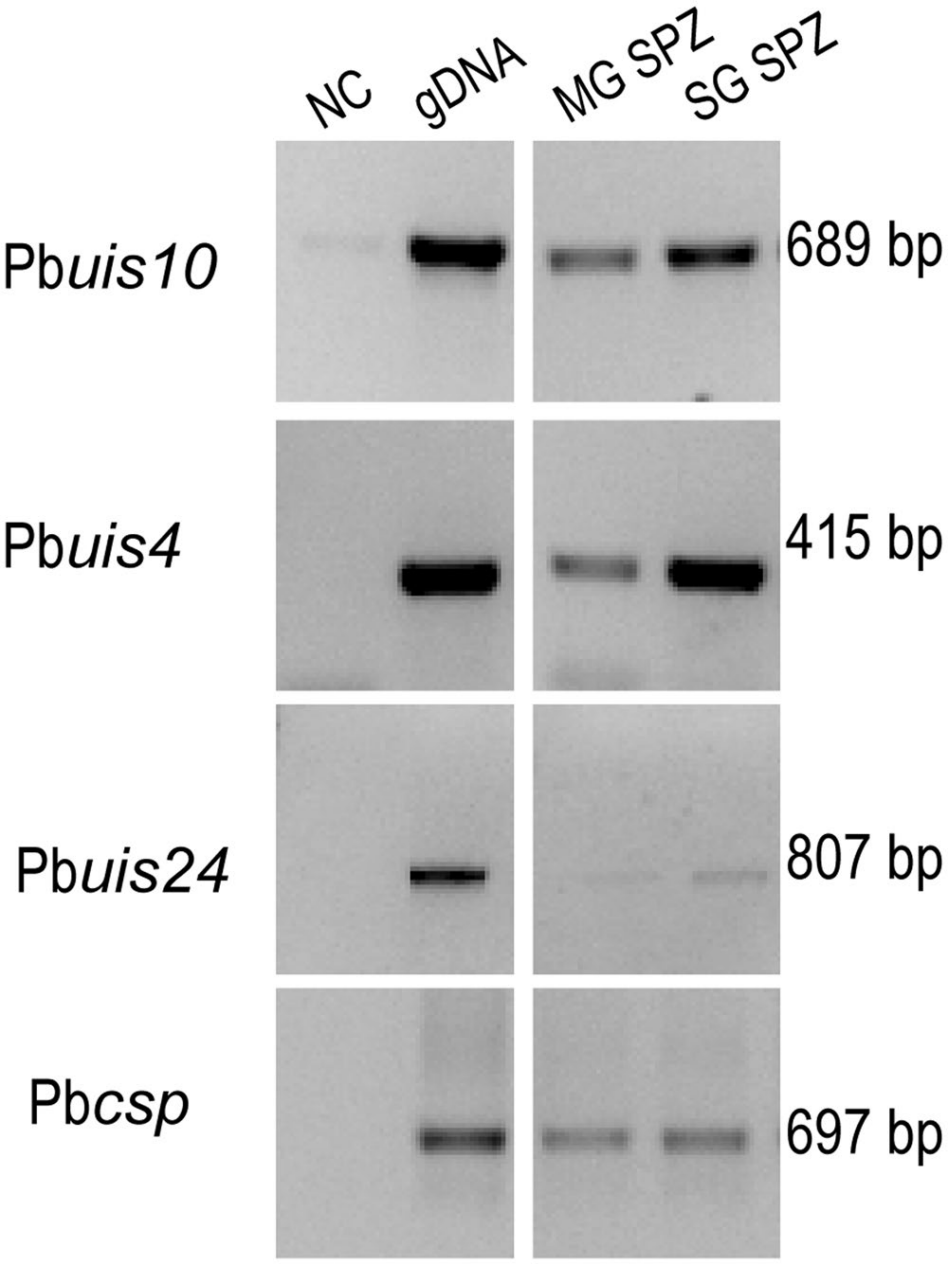
RT-PCR analysis of P.berghei Pb*uis4*, Pb*uis10*, Pb*uis24* and Pb*csp* transcripts. Templates were derived from gDNA (lane 2), dissected midguts at 11 d pbm sporozoites (lane 3, MG SPZ) and from dissected salivary glands at 21 d pbm (lane 4 SG SPZ). Lane 1 is the negative control without template (NC). The samples were amplified for 35 cycles. Molecular weight of the amplified fragments is indicated to the right. Unprocessed images of the agarose gels are shown in Supplementary Fig. S4b.

We also tested expression of the *uis* (up-regulated in infective sporozoites) genes. These genes were identified using a suppression subtractive hybridization method, which revealed the highly up-regulated transcripts in salivary gland sporozoites, as opposed to midgut sporozoites^17^. We selected the genes Pb*uis1* (serine-threonine protein kinase, PBANKA_0205800), Pb*uis2* (serine-threonine protein phosphatase, PBANKA_1328000), Pb*uis4* (early transcribed membrane protein, PBANKA_0501200), Pb*uis5* (cysteine desulfatase, PBANKA_0211300), Pb*uis24* (heat-shock protein 70, PBANKA_0914400), Pb*uis12* (RNA-binding proten, PBANKA_0506200) and Pb*uis10* (phospholipase, PBANKA_1128100). RNAseq data (www.PlasmoDB.org) indicated that the orthologues of these *P. berghei* genes in *P. falciparum* had high expression levels at the sporozoites stage. We used the Pb*csp* gene transcript as a positive control for the presence of parasites in the samples. The transcript abundance of this gene was similar in our infected *P. berghei* midgut and salivary gland samples (Fig. 2). In these experiments we retrieved samples from the PCR reactions at 25, 30 and 35 cycles to avoid saturating the reaction, and to improve quantification.

The results of the RT-PCR analysis of the *uis* gene transcripts showed that Pb*uis4* was highly expressed in the salivary gland-stage, already being detected after 25 PCR cycles. Pb*uis10* and Pb*uis24* showed a more abundant amplification product in salivary gland compared to midgut samples, but the difference was modest (Fig. 2). The other tested genes were either not expressed at the salivary gland stage, or the difference between midgut and salivary gland samples was negligible (Supplementary Fig. S1).

### Identification of *Plasmodium falciparum* salivary gland-specific transcripts

**Figure 3:**
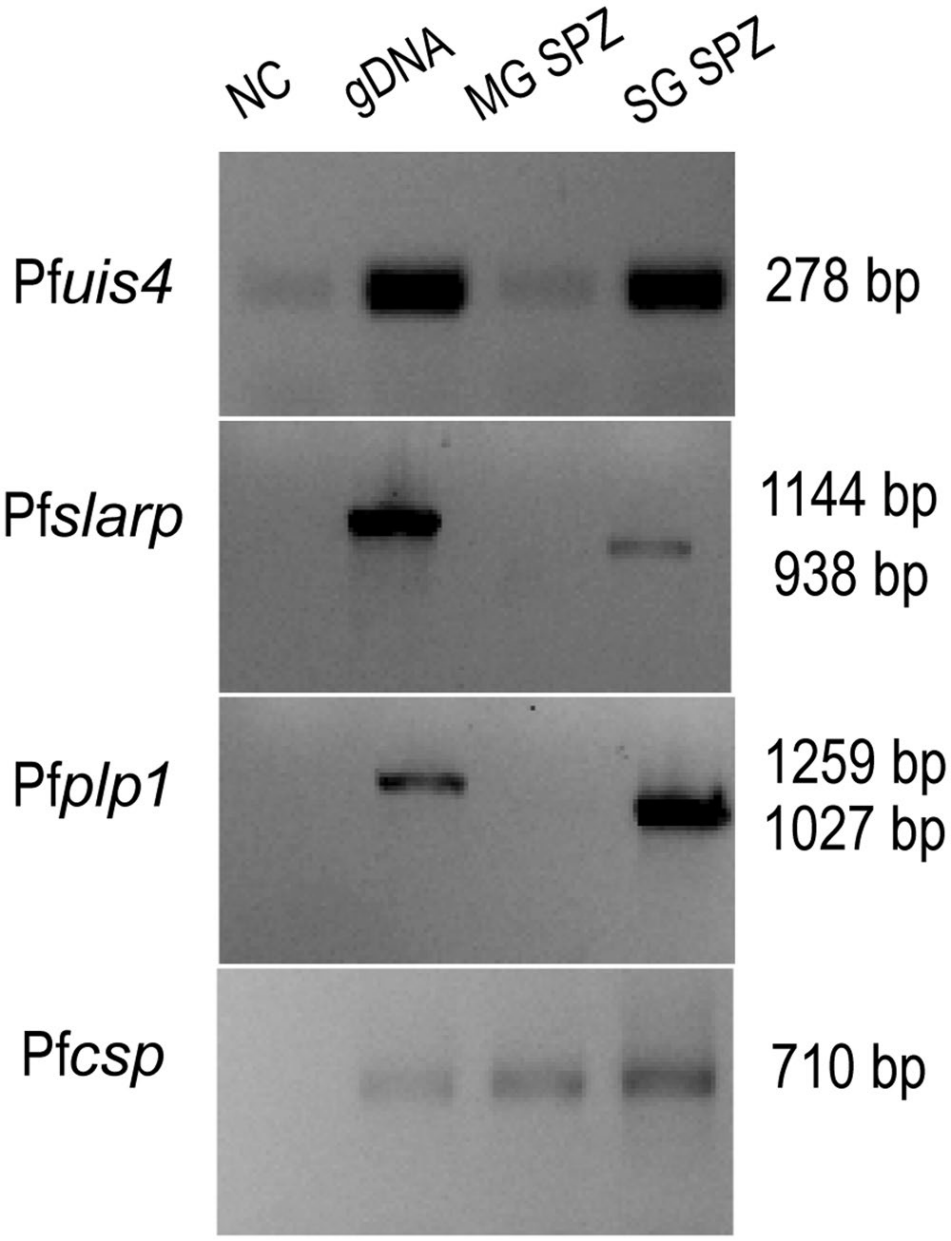
RT-PCR analysis of *P. falciparum* Pf*uis4*, Pf*slarp*, Pf*plp1*and Pf*csp* transcripts. Templates were derived from gDNA (lane 2), dissected midguts at 12 d pbm sporozoites (lane 3, MG SPZ) and dissected salivary glands at 21 d pbm (lane 4 SG SPZ). Lane 1 is the negative control without template (NC). The samples were amplified for 35 cycles. Molecular weight of the amplified fragments is indicated to the right. Unprocessed images of the agarose gels are shown in Supplementary Fig. S4c.

The above results suggested that it is possible to use RT-PCR to specifically identify mosquitoes carrying parasites in their salivary glands. To determine whether this also applied to the human parasite *P. falciparum*, we performed a similar analysis on whole infected mosquito samples that had been stored 12 or 21 days pbm. Samples from the first time point contained only midgut sporozoites inside the oocysts, while in the second time point samples contained salivary gland-invaded sporozoites. The presence of oocysts and sporozoites was confirmed by microscopic examinations of the same infected mosquito cohorts. Samples containing pools of 10 mosquitoes each were processed for RNA extraction and cDNA synthesis. Based on the *P. berghei* experiments, and *P. falciparum* sequence (Plasmodb), primers were designed and optimized for Pf*slarp*, Pf*plp1*, Pf*uis4*, Pf*uis10* and Pf*uis24*and the Pf*csp* gene was used as a positive control for the presence of parasite RNA in our samples (Supplementary Table S2). RT-PCR experiments were carried out and amplification products were analysed after different number of cycles (30, 35 and40). The results showed that Pf*uis10* was not detected in neither stage and the Pf*uis24* gene transcript was amplified at similar levels in both samples (Fig. S2). On the other hand, Pf*uis4*, Pf*plp1* and Pf*slarp* were amplified differently between the two time points (Fig. 3). This result was confirmed in a second replicate, as well as using an independent RNA preparation from the same batch of mosquitoes. However, because Pf*uis4* does not contain introns it is less suitable for assays where the isolated RNA will not be DNase-treated. On the contrary, the Pf*slarp* and Pf*plp1* genes have introns and can therefore be used in diagnostic assays that omit or cannot integrate this step.

### Development of a RT-qPCR TaqMan assay for infective mosquitoes

**Figure 4:**
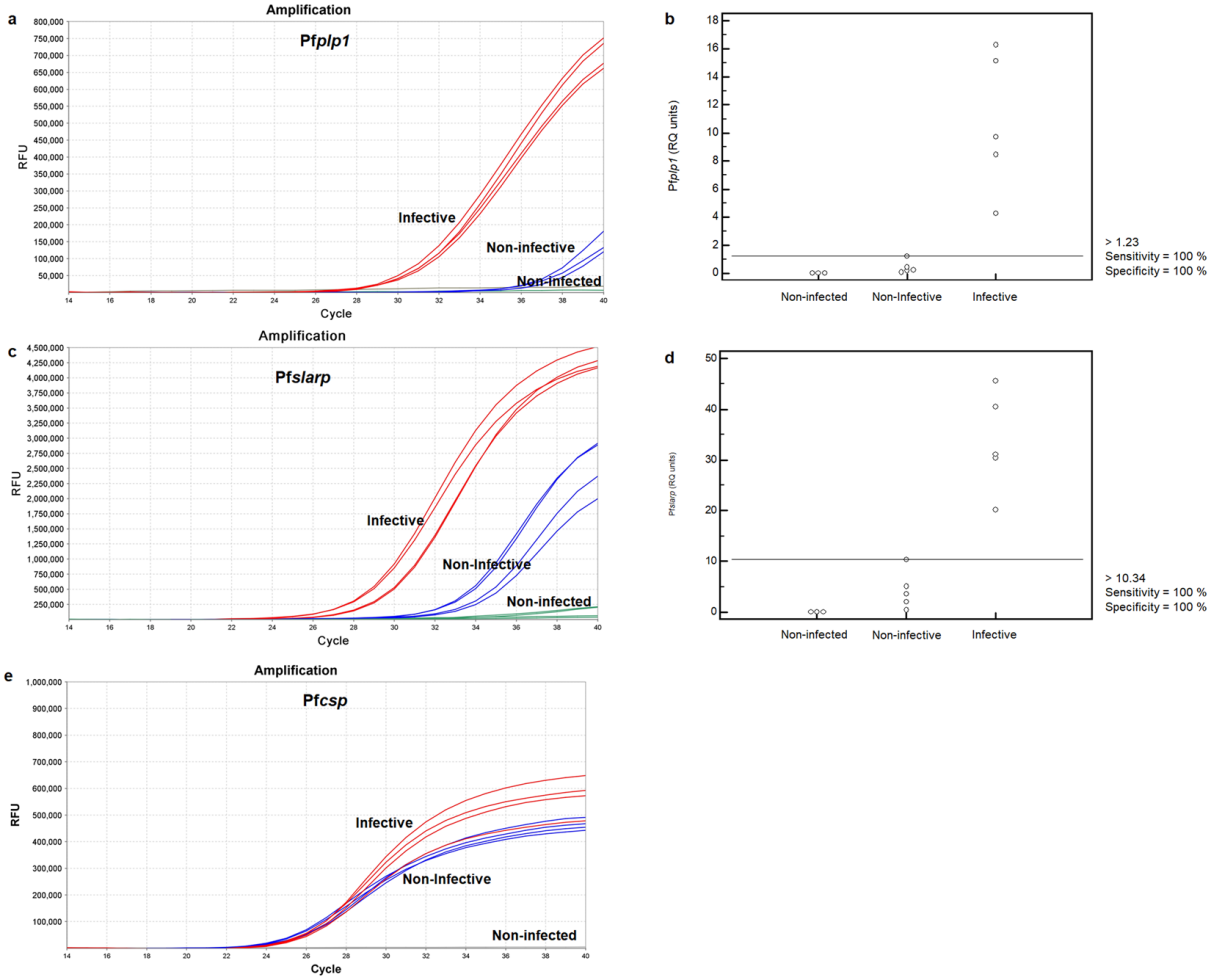
Differential diagnostic value of Pf*plp1* and Pf*slarp* expression. (**a**,**c**,**e**) TaqMan amplification curves for Pf*plp1*, Pf*slarp* and *Pfcsp genes* in infective (red color), non-infective (blue color) and non-infected (green color) samples. Four independent biological replicates are shown with two technical replicates for each curve. (**b,d**) Expression levels of Pf*slarp* and Pf*plp1* in infective, non-infective and non-infected pools.

We used our results from the gene expression analyses to develop a quantitative Reverse Transcription-real-time PCR (RT-qPCR) assay, based on TaqMan® chemistry. The gene-specific primers and probes were tested in combinations to determine optimal concentrations (Table 1). The RT-qPCR assays were used to measure Pf*slarp* and Pf*plp1*transcript abundance, normalized to Pf*csp*, in pools of whole mosquitoes. Both genes were found substantially up-regulated in the infective sample (Fig. 4a,c). We next calculated the Relative Quantification (RQ) units for each sample and target gene (RQ = 2^-dCt^, where dCt = (Ct target - Ct reference) and Ct is the threshold value).The data showed that the expression levels of Pf*slarp* were 33.5 ± 4.4 RQ units for the infective stage compared to 4.29 ± 1.7 RQ units for the non-infective pool (Table 2). Pf*plp1* expression levels were measured as 10.8. ± 2.2 at the infective stage and 0.43 ± 0.21 at the non-infective stage. By using the calculated 1.23 or 10.34 RQ units as cut-off values for Pf*plp1* and Pf*slarp*, respectively, a sensitivity and specificity of 100% can be achieved in discriminating infective from non-infective mosquitoes (Fig. 4b,d).

**Table 2:** Descriptive statistics of Pf*plp1* and Pf*slarp* gene expression in infective samples compared to non-infective and non-infected samples.

Gene	Infection status of samples	RQ (Mean ± SE)	95% CI	Ct
Pf*plp1*	Infective	10.8 ± 2.2	4.6–16.9	29.3–30.5
	Non- infective	0.43 ± 0.21	0–1.01	>37.0
	Non-infected	Undetected	Undetected	Undetected
Pf*slarp*	Infective	33.5 ± 4.4	21.3–45.8	27.1–29.6
	Non-infective	4.29 ± 1.7	0–9.01	>33.0
	Non-infected	Undetected	Undetected	Undetected

Discrimination of samples can also be more easily achieved using only Pf*slarp* or Pf*plp1* Ct values (cut-off Ct = 33.0 for Pf*slarp* and Ct = 37.0 for Pf*plp1*) without the need of calculating normalized RQ units. This is supported by the fact that Pf*slarp* Ct values ranged from 27.1–29.6 in the infective samples, compared to values greater than 33.0 in the non-infective samples. Similarly, Pf*plp1* Ct values were within the range of 29.3–30.5 in the infective samples and practically non-detectable (Ct >37.0) in the non-infective pools (Fig. 4a,c). Pf*csp* was used as a qualitative positive control rather than a quantitative normalizer in this case. Non-infected samples did not produce any Ct value.

We were able to detect Pf*slarp* and Pf*plp1* expression in single infective mosquitoes at considerably higher levels compared to non-infective ones, thus proving the applicability of the developed assay in individual mosquitoes as well (Supplementary Fig. S3).

We also investigated the sensitivity of the assay for the detection of *P. falciparum* sporozoites in the salivary glands. Infected mosquitoes were mixed with non-infected *A. gambiae* mosquitoes and then subjected to the TaqMan assay. Both Pf*plp1* and Pf*slarp* expression was unambiguously detected when the infective sample was mixed in a 1:50 or even 1:100 ratio with non-infected mosquitoes (Supplementary Table S4). By using the previously described cut-off values for gene expression it is possible to detect the infective stage of the parasite even in the 1:100 population using either of the two genes as marker.

## Discussion

The development of affordable and rapid diagnostic tests for malaria parasite infected mosquitoes, and more specifically for infective mosquitoes harbouring sporozoites in their salivary glands is of high priority for supporting evidence based vector control interventions^10^. Several assays have been devised for this purpose, including ELISA and PCR-based detection in dissected mosquito heads/salivary gland preparations^13–15,18^. Here, we report the development of a dissection-free, one-step RT-qPCR method for detecting the infective stage of *Plasmodium falciparum* in its mosquito vector.

We initially took advantage of available proteomic and RNAseq data (PlasmoDB; www.plasmodb.org). Five genes that were reported to be more highly expressed in sporozoites from the salivary glands compared to those of the midgut^19–22^ were selected. We also investigated expression of the *uis* genes which were originally identified based on the up-regulation of their transcripts in salivary gland sporozoites. Dissected midguts and salivary glands of mosquitoes infected with the rodent model parasite *P. berghei* were analysed using semi-quantitative RT-PCR. The two genes Pb*slarp* and Pb*plp1* were expressed at considerably higher levels in sporozoites from the salivary gland compared to those of the midgut (Fig. 1) and three of the *uis* genes fulfilled our selection criteria (Fig. 2). Next, we tested these genes using *P. falciparum-*infected samples. The results of this analysis suggested that transcripts of the genes Pf*slarp* and Pf*plp1* are the most suitable candidates for a RT-PCR-based assay (Fig. 3), and a RT-qPCR TaqMan assay was developed and optimized (Fig. 4).

Our results show that both selected genes present substantially higher expression levels in infective compared to non-infective pools, although each gene showed specific advantages. Pf*plp1* could be assessed in a 2-plex format together with the positive control Pf*csp*, thus minimizing time and amount of sample needed for reaction preparation (Table 1). Additionally, Pf*plp1* showed less variability between replicates compared to Pf*slarp* (Fig. 4b,d). Pf*slarp* presented generally higher expression levels but could be reliably assessed only in single-plex format without compromising sensitivity (Table 1). The use of an intron-containing *P. facliparum* housekeeping gene, instead of Pf*csp*, could also provide reliable normalization results for quantitative purposes. The assay needs to be further validated with naturally infected field-caught samples since our assays employed laboratory infected mosquitoes. However, the median infection intensity of assayed mosquitoes was 2, which is similar to levels found in field infected mosquitoes. Furthermore, our assay can only be applied with *P. falciparum-*infected samples, at present, but can conceptually be extended to other important *Plasmodium* parasites such as *P. vivax*. Future work will focus on the development of similar assays for additional epidemiologically relevant *Plasmodium* species, should the access to appropriate samples and resources become possible.

In conclusion, our novel assay represents significant improvements in detecting infective *P. falciparum* mosquitoes. The assay does not require dissection of mosquitoes nor is DNase treatment necessary. The sensitivity was high as we could detect parasite transcripts in samples containing a mix of infected with non-infected mosquitoes at a 1:100 ratio, allowing for pools of individual mosquitoes to be tested. We also found that *P. falciparum*-infected mosquitoes kept in RNAlater, at ambient temperature, for several days produced good quality RNA suitable for this assay. These are important features for the future development of diagnostic kits, given the possibility of using lyophilised RT-PCR pellets (transcriptase and probes), which can be manufactured and shipped without a cold chain and the current development of portable field-deployable real-time qPCR thermocycles. Furthermore, the assay is flexible and provides the option of multiplexing either with other parasite markers or even mosquito transcript markers such as insecticide detoxification genes (expressed primarily in abdomens) that are associated with insecticide resistance. Thus, this novel assay represents an ideal candidate for incorporation in automated diagnostics platforms for mosquito vector surveillance^10^.

## Electronic supplementary material

Supplementary Information
