# Supplementary material for: New rapid one-step PCR diagnostic assay for Plasmodium falciparum infective mosquitoes
Source: Sci Rep. 2018 Jan 23;8:1462. doi: 10.1038/s41598-018-19780-6 (PMC5780459; doi:10.1038/s41598-018-19780-6)
Supplement: Supplementary file 1 — Supplementary Information [file 41598_2018_19780_MOESM1_ESM.pdf]

New rapid one-step PCR diagnostic assay for *Plasmodium falciparum* infective mosquitoes

Mary Kefi[1,2], Konstantinos Mavridis[1], Maria L.Simões[3], George Dimopoulos[3], Inga Siden-Kiamos[1*], John Vontas[1,4]

[1]Institute of Molecular Biology and Biotechnology, Foundation for Research and Technology-Hellas, Heraklion 70013, Greece

[2]Department of Biology, University of Crete, VassilikaVouton, Heraklion 70013, Greece

[3]W. Harry Feinstone Department of Molecular Microbiology and Immunology, Johns Hopkins Bloomberg School of Public Health, 615 N Wolfe St., Baltimore, MD 21205, USA.

[4]Pesticide Science Laboratory, Department of Crop Science, Agricultural University of Athens, 11855 Athens, Greece

Table S1. Primer sequences and main PCR conditions for the thirteen *P. berghei* genes tested.

Gene	Forward primer (5´-3´)	Reverse primer (5´-3´)	Amplicon size gDNA(bp)	Amplicon size transcript (bp)
Pb*uis1*	GGATGGGCAAAGACAAAGAA	TCACCTATTGTGCTCCACCA	**726**	**726**
Pb*uis2*	CCCGAGGTAGATGAATTGGA	GCTCACGGAAGGATCGATAA	**622**	**622**
Pb*uis4*	CCCATTGATGAGACAAACGA	ATGCTTCTTCAGTGGGGCTA	**415**	**415**
Pb*uis5*	GGTTGGGAATCAGAACAAGC	TGCTGAACCCGAACTTAGTG	**870**	**870**
Pb*uis10*	TGCAGAGGGTACTGTTGACG	TCGAACCAGCAAATGGTACA	**689**	**689**
Pb*uis12*	TGCAGAAGACAGCGAATTGT	TCTTCCCGTTTTTCCAACAC	**816**	**816**
Pb*uis24*	GGAAGGAAAGCAAGGAAAGG	GGTCCTGTTTGATTGGCAGT	**807**	**807**
Pb*csp*	AGCATCCAAGCCCAAAGG	CCGCGCTTGGGATATAAG	**697**	**697**
Pb*spect1*	AAGCATTGAACCAAAAGGAA	TTTTTGCTTCTCCTTTTCCA	**683**	**455**
Pb*slarp*	TGAACCCAAATGATCAAGCA	GAATCGGCACAAGGCACTAT	**874**	**773**
Pb*gest*	TAATTCCCTCGGGAACAAGC	TTGGTGGCTTCCAATGTTTT	**835**	**480**
Pb*plp1*	CAAGCGTAGGGGGATCTACA	CTTTCCCCGATGAAGATGAA	**898**	**698**
Pb*spatr*	CCTGATGTTGGTGCAGACAC	TCCAATCCGACCAAGGACTA	**793**	**457**

Table S2. Primer sequences and main PCR conditions for the six *P. falciparum* genes tested.

Gene	Forward primer (5´-3´)	Reverse primer (5´-3´)	Amplicon size gDNA(bp)	Amplicon size transcript (bp)
Pf*uis4*	ATGAAGGTTTCTAGGCATACCG	GTCGGATCCATCATTCACCT	**278**	**278**
Pf*uis10*	TTTAGCGAGTTTTCGGAGGA	CATCAGCGGGGAAATTCTTA	**865**	**865**
Pf*uis24*	TGTGAATCCTGACGAAGCTG	GGGCTCCTTGTTGAGCATT	**743**	**743**
Pf*csp*	CGGATGGTAATCCTGATCCA	ACATGGGGACCATTCAGTTG	**710**	**710**
Pf*slarp*	GAATGATCTTAATTCCAATGAGC	CAGCCCTGGTATATAAATTACTGTC	**1144**	**938**
Pf*plp1*	AGTGCTGGAGGATCTACTGATG	CATGTCCAGAACCATGAACAG	**1259**	**1027**

Table S3. MIQE checklist*

Category	Item to be described/detailed	Location in Manuscript (Page No.)	Comments by Author
**Sample**	Type (blood, etc.)	Materials and Methods (p 5-6)	
	Method of dissection/procurement	Materials and Methods (p 5-6)	
	Processing procedure	Materials and Methods (p 5-6)	
	If frozen, how and how quickly?	Materials and Methods (p 5)	
	If fixed, with what and how quickly?	-	not applicable
	Storage conditions and duration	Materials and Methods (p 5)	
**Extraction**	Method or instrument	Materials and Methods (p 6-7)	
	Reagents/kits/modifications	Materials and Methods (p 6-7)	
	DNAse or RNAse treatment	-	not applicable, DNA was tested as a template and was not amplified
	Evidence for lack of contamination (DNA or RNA)	Materials and Methods (p 6-7)	
	Nucleic acid quantification	Materials and Methods (p 7)	
	RNA integrity	Materials and Methods (p 7)	
**Reverse Transcription**	Complete reaction conditions, including all components and their concentrations	Materials and Methods (p 6-7) and Table 1	
	RNA amount and reaction volume	Materials and Methods (p 6-7)	
	Priming oligo sequence(s)	Table 1	
	Cqs with and without reverse transcriptase	-	not applicable, DNA was tested as a template and was not amplified
**qPCR target**	HUGO gene abbreviation	-	not applicable
	Sequence accession number	Materials and Methods (p 7)	
	Amplicon length	Table 1	
	In silico specificity (BLAST)	Materials and Methods (p 7)	
	Location by exon/intron	Materials and Methods (p 7)	
	Identify the splice variants amplified	Materials and Methods (p 7)	
	All primer/probe sequences	Table 1, Table S1, Table S2	
	Location and identity of any oligonucleotide modifications	Table 1	
**qPCR protocol**	Complete reaction conditions, including all components and their concentrations	Materials and Methods (p 7-8)	
	cDNA/DNA amount and reaction volume	Materials and Methods (p 7-8)	RNA was used as template
	Instrument identification and complete thermocycling parameters	Materials and Methods (p 7-8)	
**qPCR validation**	Evidence for PCR specificity (gels, sequencing, or melting curves)	Table 1 (probe sequences)	TaqMan chemistry
	Template inhibition data (template titrations)	Table 1	
	For SYBR Green I reactions, the Cq of the no template control	-	not applicable

	Calibration curves with slope and intercept	Table 1	
	PCR efficiency from the slope	Table 1	
	r2 of the calibration curve	Table 1	
	Evidence for the linear dynamic range	Table 1	
	Evidence for the limit of detection	Table 1	
	For multiplexed assays, the efficiency and limit of detection of each assay	Table 1	
**Data analysis**	qPCR analysis method/software	Materials and Methods (p 8)	
	Method of Cq determination	Materials and Methods (p 8)	
	Results of no template controls	Materials and Methods (p 7-8)	
	Justification of number and choice of reference genes	Materials and Methods (p 7)	
	Normalization method	Materials and Methods (p 8)	
	Number and stage (reverse transcription or qPCR) of technical replicates	Materials and Methods (p 8)	
	Intra-assay variation in terms of concentration, not Cq	Table 1	
	Statistical methods/software	Materials and Methods (p 8)	

*according to “The MIQE guidelines: minimum information for publication of quantitative real-time PCR experiments. Clin Chem. 2009 Apr;55(4):611-22.”

Table S4. Detection of Pf*plp1* and Pf*slarp* genes in mixed populations of infective and non-infected mosquitoes (N=2 biological replicates used for each population).

Gene	Population	RQ (Mean ± SE)	Ct (Mean ± SE)
Pf*plp1*	1 infective and 9 non-infected mosquitoes (1:10)	46.7 ± 1.6	25.90 ± 1.0
	1 infective and 49 non-infected mosquitoes (1:50)	26.4 ± 12	26.10 ± 0.87
	1 infective and 99 non-infected mosquitoes (1:100)	21.1 ± 6.0	32.8 ± 0.03
Pf*slarp*	1 infective and 9 non-infected mosquitoes (1:10)	85.0 ± 58	25.5 ± 0.22
	1 infective and 49 non-infected mosquitoes (1:50)	55.0 ± 14	26.5 ± 1.0
	1 infective and 99 non-infected mosquitoes (1:100)	29.2 ± 11	32.28 ± 0.10

Input amount RNA in these experiments was 10x higher than in experiments with pools of 10 mosquitoes.

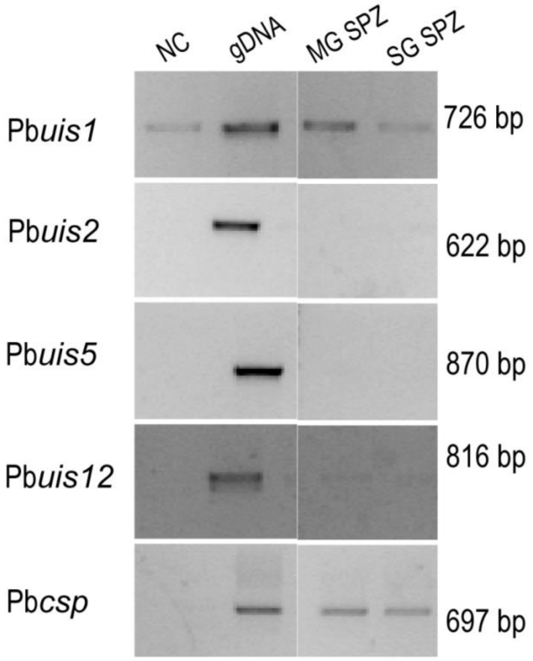

Figure S1. RT-PCR analysis of *P.berghei* Pb*uis1*, Pb*uis2*, Pb*uis5* and Pb*uis12* transcripts. Templates were derived from gDNA (lane 2), dissected midguts at 11 d pbm sporozoites (lane 3, MG SPZ) and dissected salivary glands at 21 d pbm (lane 4 SG SPZ). Lane 1 is the negative control without added template (NC). The samples were amplified for 35 cycles. Molecular weight of the amplified fragments is indicated to the right. Unprocessed images of the agarose gels are shown in Supplementary Fig. S4d.

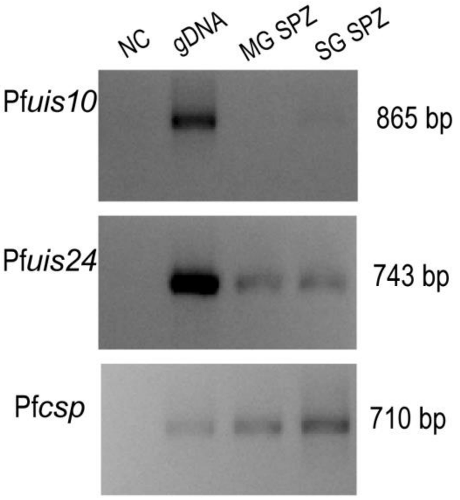

Figure S2. RT-PCR analysis of *P. falciparum* Pf*uis10* and Pf*uis24* transcripts. Templates were derived from gDNA (lane 2), dissected midguts 12 d sporozoites (lane 3, MG SPZ) and dissected salivary glands at 21 d pbm (lane 4 SG SPZ). Lane 1 is the negative control without added template (NC). The samples were amplified for 35 cycles. Molecular weight of the amplified fragments is indicated to the right. Unprocessed images of the agarose gels are shown in Supplementary Fig. S4e.

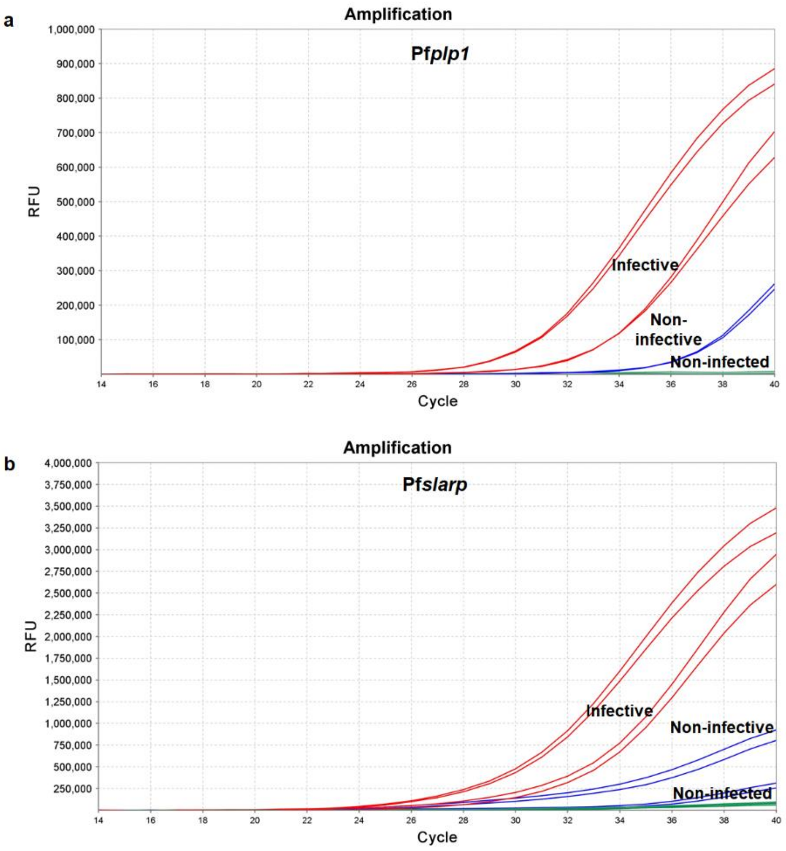

Figure S3. Detection of Pf*plp1* (a) and Pf*slarp* (b) expression in single infective (red color) and single non-infective (blue color) mosquitoes. Two different mosquitoes are shown with two technical replicates for each sample. No expression was detected in non-infected mosquitoes (green color).

a.

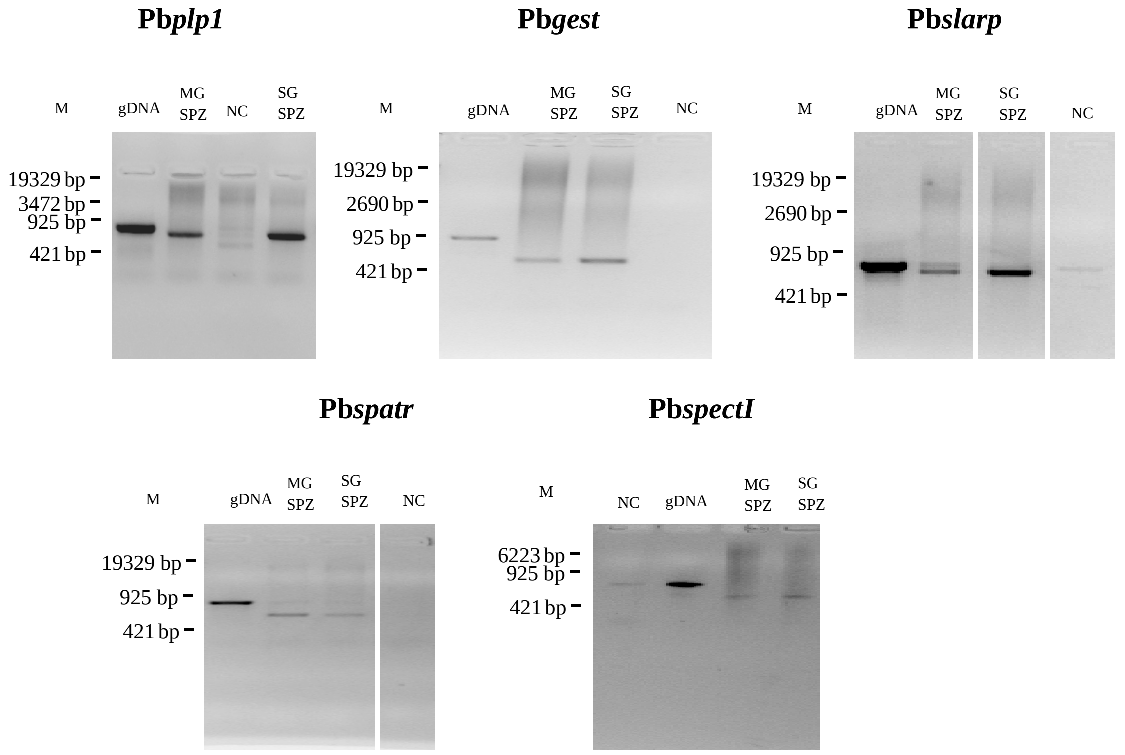
Pbplp1
Pbgest
Pbslarp
M gDNA MG SPZ NC SG SPZ
M gDNA MG SPZ SG SPZ NC
M gDNA MG SPZ SG SPZ NC
19329 bp
3472 bp
925 bp
421 bp
19329 bp
2690 bp
925 bp
421 bp
19329 bp
2690 bp
925 bp
421 bp
Pbspatr
PbspectI
M gDNA MG SPZ SG SPZ NC
M NC gDNA MG SPZ SG SPZ
19329 bp
925 bp
421 bp
6223 bp
925 bp
421 bp

b.

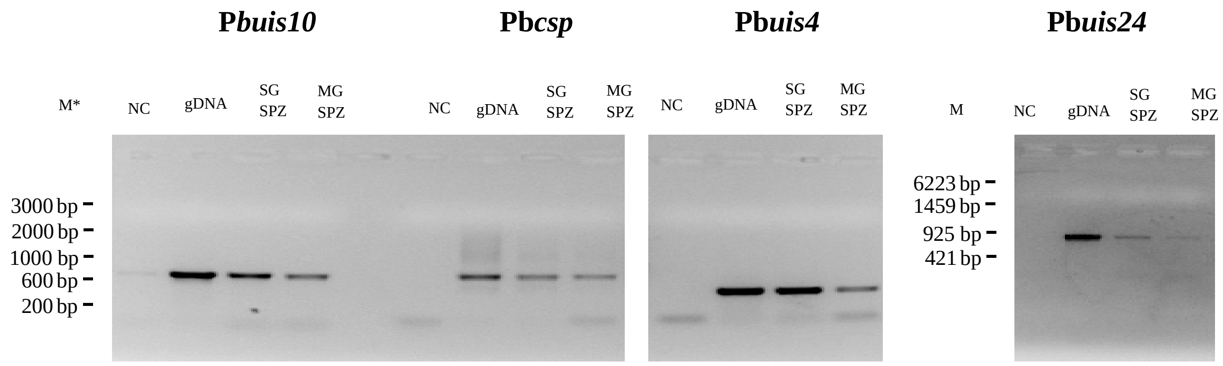
Pbuis10
Pbcsp
Pbuis4
Pbuis24
M* NC gDNA SG SPZ MG SPZ
NC gDNA SG SPZ MG SPZ
NC gDNA SG SPZ MG SPZ
M NC gDNA SG SPZ MG SPZ
3000 bp
2000 bp
1000 bp
600 bp
200 bp
6223 bp
1459 bp
925 bp
421 bp

c.

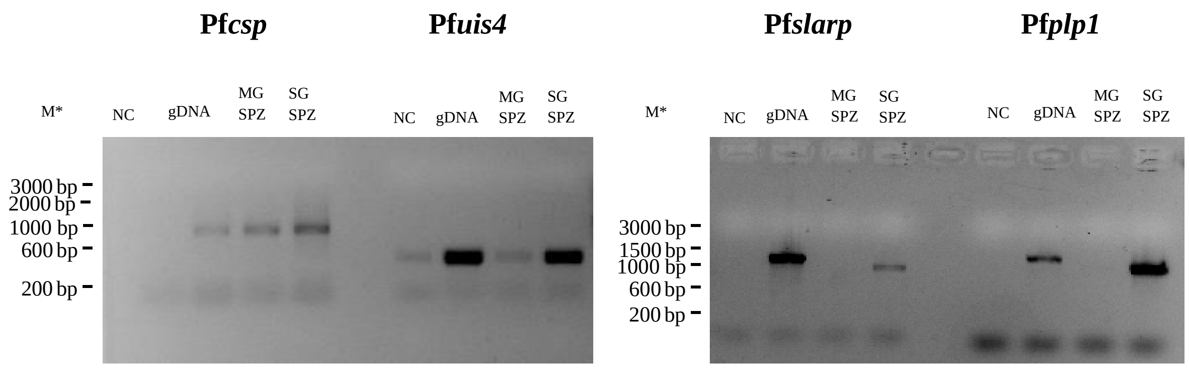
Pfcsp
Pfuis4
Pfslarp
Pfplp1
M* NC gDNA MG SPZ SG SPZ
NC gDNA MG SPZ SG SPZ
M* NC gDNA MG SPZ SG SPZ
NC gDNA MG SPZ SG SPZ
3000 bp
2000 bp
1000 bp
600 bp
200 bp
3000 bp
1500 bp
1000 bp
600 bp
200 bp

d.

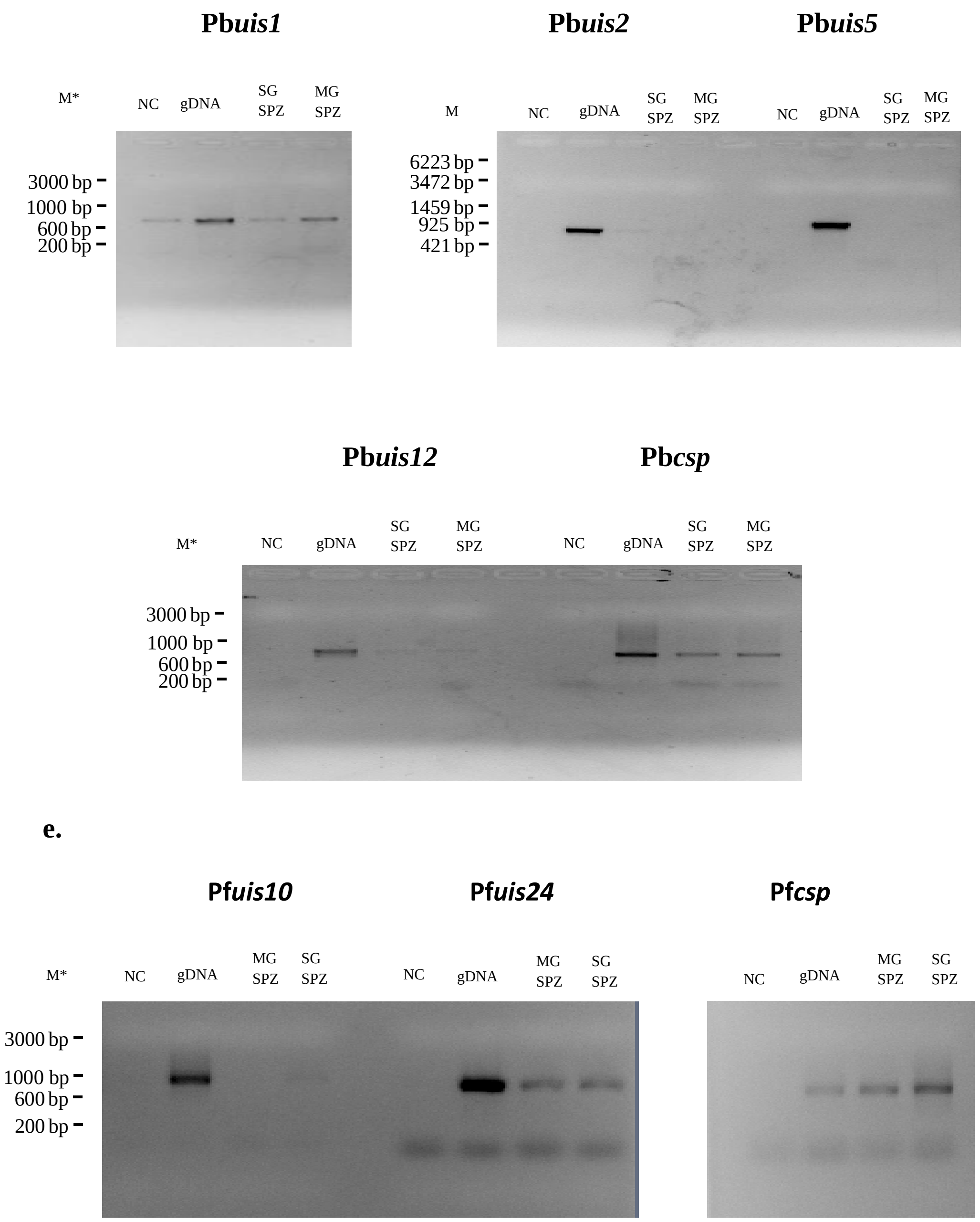

Fig. S4. Unprocessed images of agarose gels shown in Figs. 1 (a), 2 (b), 3 (c), and Supplementary Figs. S1 (d) and S2 (e). M: Phage lambda DNA digested with StyI , M*: 100bp DNA ladder.
